# Biosynthesis of novel metallic silvers on kraft papers using *cephalotaxus harringtonia* fruit extract as a sustainable stabilizing agent (KP@AgNP)

**DOI:** 10.3389/fbioe.2022.967166

**Published:** 2022-08-10

**Authors:** Shaofeng Wei, Xiaoyi Liu, Jiao Xie, Huijuan Liu, Qibing Zeng, Guoze wang, Peng Luo

**Affiliations:** The Key Laboratory of Environmental Pollution Monitoring and Disease Control, Ministry of Education, School of Public Health, Guizhou Medical University, Guiyang, China

**Keywords:** green silver nanoparticles synthesis, *Cephalotaxus harringtonia* fruit extracts, kraft paper, functionalization, UV-protection

## Abstract

Greenly synthesized silver nanoparticles (AgNPs) on different cellulosic materials show tremendous potential for colorful, biocidal, and reasonably strong products by replacing the traditional chemical-based synthesis protocols. This study reports on a novel *in situ* synthesis protocol for synthesizing green and sustainable AgNPs over cellulosic kraft paper substrates using a bio-based stabilizing agent (*Cephalotaxus harringtonia* fruit extract). The protocol could play a significant role in packaging industries. The aqueous extracts of *Cephalotaxus harringtonia* fruits have been used to synthesize the metallic silver. The deposited AgNPs values were investigated through XRF (X-ray fluorescence) analysis. The number of deposited nanoparticles (NPs) was 268 ± 7, 805 ± 14, and 1,045 ± 16 PPM, respectively for 0.5, 1.5, and 2.5 mm silver precursors. The developed products were tested with SEM (scanning electron microscopy), SEM-mediated elemental mapping, EDX (energy disruptive X-ray), FTIR (Fourier transform infrared spectroscopy), and XRD (X-Ray diffraction). XRD analysis further confirmed the presence of peaks for elemental AgNP on the deposited papers. Colorimetric values were measured to confirm the colorful appearances of the developed metallic silvers. Mechanical properties were tested in terms of the tensile index and bursting index. Moreover, the statistical analysis of coefficient of variations (R^2^) and a post-hoc ANOVA test that adopted the Newman-Keul methodology also confirm the significance of developed nanoparticles in the papers. The shielding capacity against UV light was also investigated; all the AgNPs-treated products provided values higher than 40, demonstrating the strong UV resistance capability of the kraft paper material. Overall, the study confirms a successful development of green AgNPs on paper materials.

## 1 Introduction

Due to their unique properties, AgNPs have many potential applications in sustainable coloration, functionalization, and food packaging. Greenly synthesized AgNPs exhibit antiviral and anti-inflammatory properties. They also add thermal stability, improved mechanical performance, and colorful appearances to the deposited products (Abou El-Nour et al., 2010; Ibrahim, 2015; Kedi et al., 2018). Versatile methods like chemical and biological approaches generate green nanoparticles from metallic salts. However, traditional chemical-based synthesis methods were not always safer because some toxic reducing agents were used to synthesize metallic AgNPs (Khatami et al., 2019); hence, naturally derived plant materials have been receiving increasing attention. Plants are a natural treasure of materials with important phytochemical components, and the green synthesis of metallic silver from plant materials is also termed phytofabrication. Plant derivatives are abundant in nature, but the beneficial effects of most derivatives have yet to be investigated. Among these beneficial effects, nanoformulations provide improved functionalities in the forms of mechanical properties, thermal stabilities, and UV resistance. Therefore, plant-based stabilizing/capping agents are used for green AgNPs synthesis—which are less labor intensive in preparing nanocolloids and associated products and, consequently, save time, cost, and energy consumption—are becoming a superior alternative solution to chemical-based reducing agents (Cittrarasu et al., 2021). Until 2009, nearly 500 tonnes of AgNPs were produced throughout the globe (estimated), whereas an increase in upto 900 tonnes is expected by 2025 (approximately) (Islam et al., 2021).

Nowadays, experiments on various plant extracts like leaves, stems, heartwoods, flowers, roots, and seeds are being conducted to reduce and stabilize metallic salts. Some of these extracts have included *Thespesia lampas* pant (Ashok et al., 2018), *Lythrum salicaria* extract (Mohammadalinejhad et al., 2019), chitosan/date seed extract (Ramadan et al., 2020), *Ocimum sanctum* leaf (Kulkarni and Kulkarni, 2013), *Gymnema sylvestre* (Heera et al., 2015). However, to our knowledge, using *Cephalotaxus harringtonia* fruits to stabilize and reduce silver precursors to produce green AgNPs has not yet been reported. The current research study explores the possibility of green synthesis of metallic silvers using *Cephalotaxus harringtonia* seed aqueous extracts. The reason for selecting *Cephalotaxus harringtonia* fruit is its tremendous medical applications, including anticancer, internal bleeding, cough, hookworm disease treatment, anti-inflammatory, antimicrobial, antioxidant, immunomodulatory, and so on (Da-Cheng et al., 2021). Additionally, various components like flavonoids, phenolics, lignans, sesquiterpenoids, and diterpenoids are present in *Cephalotaxus* species (Da-Cheng et al., 2021; Li et al., 2021), which also facilitate the synthesizing of metallic silver. Moreover, *Cephalotaxus* species have also gained attention for pharmacokinetics and drug metabolism. Therefore, the current study selected the extract of the *Cephalotaxus* species to functionalize kraft paper materials for potential food packaging applications.

Concerns about sustainable packaging materials production in terms of biodegradable films and coatings has increased in the food packaging industry. Food packaging coatings are extremely important because they provide a protective physical barrier during transportation and storage, thereby minimizing food waste and negative environmental consequences (Nechita et al., 2020). However, bioplastic materials have begun replacing paper-based materials because paper materials do not guarantee the same level of protection against bacterial or fungal contamination. Bacterial and fungal-resistant packaging materials are essential for food products. AgNPs coatings on paper surfaces could improve these materials and motivate food manufacturers to continue using paper-based packaging materials, which would reduce the environmental burdens caused by plastic-based petroleum-generated products. Moreover, foods will also be safeguarded from external bacterial attacks. The cellulosic materials could be coated with greenly synthesized metallic silvers with improved mechanical properties, which will keep the foods safer from bacteria and fungi.

Plant-based synthesis protocols are eco-friendly and sustainable. They are also safer, quicker, biocompatible, non-hazardous, and reliable compared to the traditional chemical-based synthesis of metallic silver. Additionally, cellulosic kraft paper materials are also biocompatible and sustainable. To the best of our knowledge, no research to functionalize kraft papers using *Cephalotaxus harringtonia* fruit extract as the stabilizing agent has been conducted yet. Coated kraft papers can be used as prominent materials for food packaging industries. Coating paper surfaces with green AgNPs offers the food packaging community a new milestone that could provide improved mechanical properties, UV protection, sustainability, and durable, colorful appearances.

## 2 Materials and methods

### 2.1 Materials

Bleached cellulosic kraft papers that were 0.15 mm thick and weighed 120 g/m^2^ were collected from the local markets of Guizhou, China and prepared to 50 mm × 50 mm dimensions before undergoing nanosilver treatments. Metallic salt (AgNO_3_) was procured from Sigma Aldrich Co., St. Louis, United States. *Cephalotaxus harringtonia* fruit was collected from the local forested areas of Guizhou, China. The fruit was processed before the aqueous extraction preparations in the laboratory.

### 2.2 Methods

#### 2.2.1 Aqueous extraction of *Cephalotaxus harringtonia plants* fruits

The collected *Cephalotaxus harringtonia* fruit was washed with distilled water to remove debris and dust that adhere to the surfaces when the fruit is grown in nature. The washed fruit was then dried in a laboratory dryer for 60 min at 90°C. Afterwards, the dried fruit was crushed with crushing equipment to create the particles needed to facilitate the extractions. The aqueous solutions of *Cephalotaxus harringtonia* fruits were prepared from the crushed particles. To produce aqueous solutions of *Cephalotaxus harringtonia* fruit (20% w/v), we mixed 80 g of the crushed dried fruit into a beaker containing 400 ml of distilled water. The mixture was then heated at 90°C for 60 min. The boiled solution was then filtered using filter paper to remove the boiled fruit particles from the solutions for successful extractions. The beakers containing extractions were then sealed and put into a refrigerator at 4°C for future use. The method also goes in line with other cellulosic materials coating and functionalization using green AgNPs (Aladpoosh et al., 2014).

#### 2.2.2 Green synthesis of metallic silver

**FIGURE 1:**
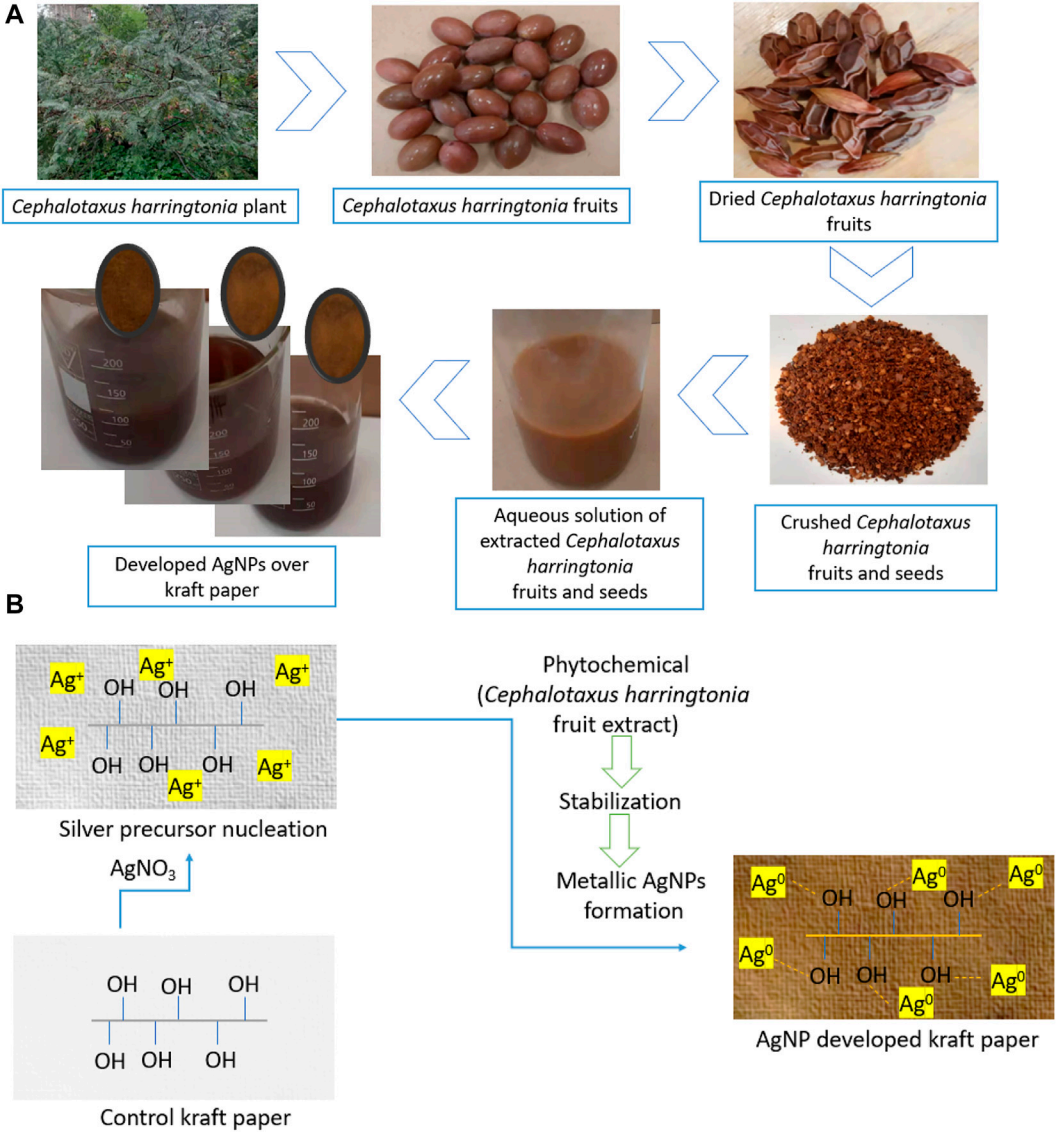
**(A)** A schematic representation of AgNPs depositions over paper and **(B)** Green AgNPs development over cellulosic kraft paper material.

To synthesize green metallic silver, we mixed silver precursor and the aqueous extracts in the beakers containing distilled water and kraft paper. Initially, AgNO_3_ was poured into the beaker and stirred continuously to mix them well. Later, the papers were soaked with distilled water and put in the beakers containing AgNO_3_ solutions. The aqueous solutions were dripped dropwise into the beakers, and the color of the solutions started to change from a milky white to light yellow/brownish, confirming the successful synthesis of metallic silver in the synthesis bath. This color change is in line with other researchers (Zuas et al., 2014; Aryan et al 2021). Finally, the beakers prepared for 0.5, 1.5, and 2.5 mM (Table 1) AgNO_3_ precursor and the *Cephalotaxus harringtonia* fruit aqueous extracts were heated at 80°C for 50 min to produce the nanosilver coating over the kraft paper substrates. The samples were named P@Ag1, P@Ag2, and P@Ag3, respectively and the control was labelled P@C. Figure 1 depicts the detailed extraction procedure and AgNPs development over the kraft paper. The paper samples were washed and rinsed thoroughly at ambient temperature (25°C) after NPs coating to remove the unattached nanoseeds from the surfaces. A 15 min drying process at 80°C in the laboratory drier followed. A similar *in situ* synthesis protocol for synthesizing green AgNPs over cellulosic cotton fabric was reported by other researcher (Maghimaa et al., 2020).

**TABLE 1:** Recipe for nanosilver coating over paper substrates.

Sample	Concentration of AgNO_3_ (mm)	Fruit extract (MW in %, v/v)
P@C	0	0
P@Ag1	0.5	3.5
P@Ag2	1.5	3.5
P@Ag3	2.5	3.5

#### 2.2.3 Characterizations adopted for AgNPs coating on kraft papers

The quantitative values of AgNPs concentrations deposited on kraft paper substrates were characterized using an XRF analyzer. The morphological images were captured using an SEM instrument (S 3400 N, Tokyo, Japan) at 8.00 kV. SEM-mediated EDX and elemental mappings images were also investigated further. We used a special software (Quantax Esprit 1.9, Berlin, Germany) to continue with EDX/elemental mapping analysis. The chemical bonding between the paper materials and AgNPs was investigated further using FTIR analysis (4,000–400 cm^−1^). The instrument used for FTIR analysis was FT/IR-6300 (Jasco, Japan). Moreover, we performed the spectroscopic analysis using a Konica Minolta instrument (CM-2600d, Japan) under D65/standard daylight within wavelengths ranging from 400 to 700 nm. The spectroscopic analysis provided the color values of the control and AgNPs-treated products. The color fastness properties of the papers were assessed according to the ISO 105-B02 standard. The XRD analysis was recorded using an X’pert Pro MDP X-ray diffractometer (PANalytical, Almelo, Netherlands). The applied voltage during the XRD test was 40 kV and the current was 30 mA. The samples were tested within 4°–82° (2θ) range. Moreover, the solar UV-protection factor was determined using a Startek UV protection application software (version 3.0, Startek technology) for both the control and AgNPs deposited fabrics. Thermal stability of the control and AgNPs-treated materials were tested by Themys thermal analyzer (Setaram Instrumentation, France) within 25–825°C.

## 3 Results and discussions

### 3.1 Mechanisms behind the green nanosilver synthesis

This study used the sustainable capping agent to synthesize the green metallic silver from AgNO_3_ by adopting an *in situ* synthesis protocol. Generally, the color of the nanocolloid starts to change from a milky to yellowish/yellowish-brown when the aqueous extract solutions are added to the beaker containing AgNO_3_ (Parvataneni, 2020) as the Ag^+^ is reduced to metallic silver (Ag^0^) due to photocatalytic actions (Eq. 1) (Eustis et al., 2006). But after a few minutes of mixing, the color turns brownish and assumes a darker brownish tone. Figure 1B displays the detailed formation mechanism of metallic silver.

$$\mathrm{Cellulosic}\;\mathrm{paper}-{\text{OH}+\text{Ag}}^{+}{\text{NO}}_{3}^{\text{\_}}\to \mathrm{Cellulosic}\;\mathrm{paper}{-\text{O}}^{-}{-\text{Ag}}^{{+}^{{\text{e}}^{-}}}\to \mathrm{Cellulosic}\;\mathrm{flax}{-\text{O}}^{-}{-\text{Ag}}^{\text{0}}$$ (1)

#### 3.2 Quantitative analysis of greenly synthesized AgNPs

**FIGURE 2:**
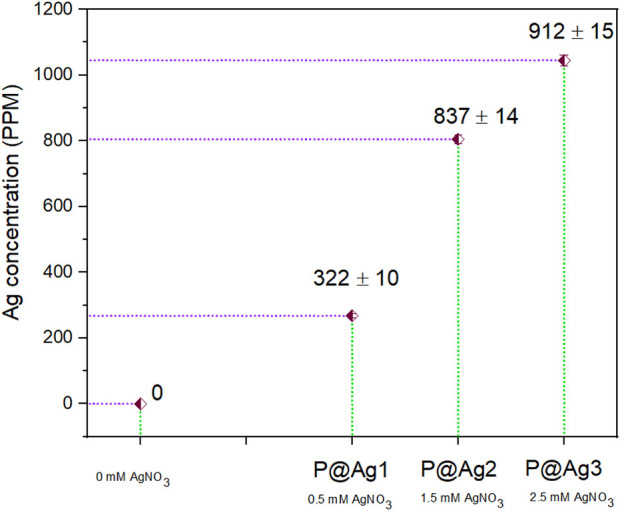
Nanosilver content measured by XRF test.

The quantification of AgNPs on deposited *kraft paper* is one of the significant factors that confirm and determine the developed particles on the substrates. The higher the silver precursor used in the nanocolloid system, the higher the deposited nanoparticles. Therefore, an XRF test was conducted to analyze the nanosilver content on the solid samples; as expected, the control sample showed no metallic particles. The PPM values were as follows: P@Ag1, 322 ± 10; P@Ag2, 837 ± 14; and P@Ag3, 912 ± 15 (Figure 2). Interestingly, it is possible to regulate and tune AgNPs development in the *in situ* synthesis system. Moreover, the XRF analysis data were further verified in terms of statistical tools using coefficient of variations. The R^2^ value (0.91) found for XRF tests against different loadings of silver precursor demonstrates that AgNO_3_ has a strong relationship in enhancing the developed AgNPs over the paper surfaces.

### 3.3 Morphological characterization of synthesized AgNPs on paper

**FIGURE 3:**
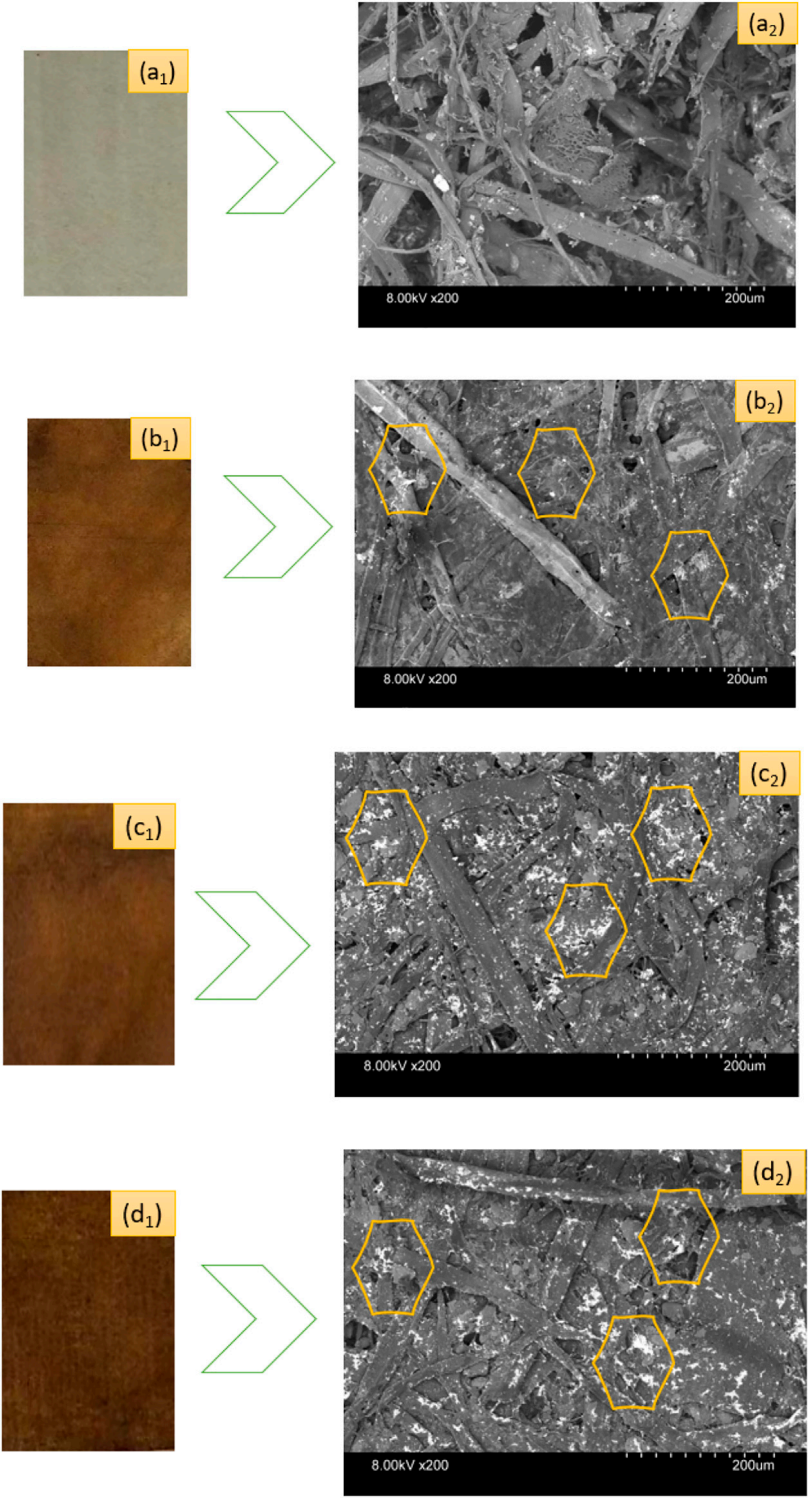
Photographs of control and nanosilver deposited kraft paper samples: **(A**_**1**_ **)** P@C, **(B**_**1**_ **)** P@Ag1, **(C**_**1**_ **)** P@Ag2, and **(D**_**1**_ **)** P@Ag3. SEM images of control and nanosilver deposited kraft paper samples: **(A**_**2**_ **)** P@C, **(B**_**2**_ **)** P@Ag1, **(C**_**2**_ **)** P@Ag2, and **(D**_**2**_ **)** P@Ag3.

Figure 3 depicts the SEM micrographs showing the existence of nanosilver clusters on the coated papers. However, the control kraft paper samples did not exhibit the presence of nanosilver as the nanocoated test specimens did. The presence of AgNP clusters is displayed using yellow-colored benzene rings in the respective SEM images. We found that the phytochemicals mediated and developed AgNPs display spherical microstructural shapes. Other researchers have made similar observations in plant-mediated biosynthesis protocol (Ibrahim, 2015). However, sample 2 and sample 3 exhibited more nanoparticles than sample 1. It seems the nanosilvers display heavier clusters with the increase in nanosilver contents.

**FIGURE 4:**
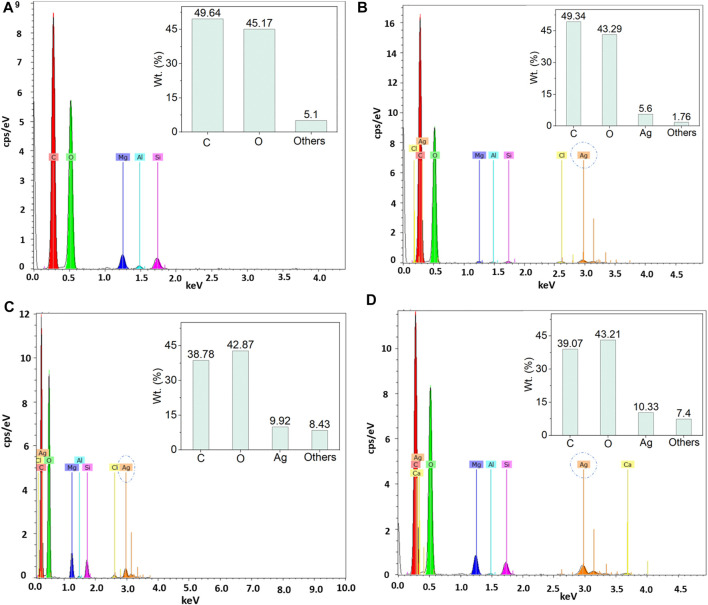
EDX analysis of control and nanosilver deposited kraft paper samples: **(A)** P@C, **(B)** P@Ag1, **(C)** P@Ag2, and **(D)** P@Ag3.

**FIGURE 5:**
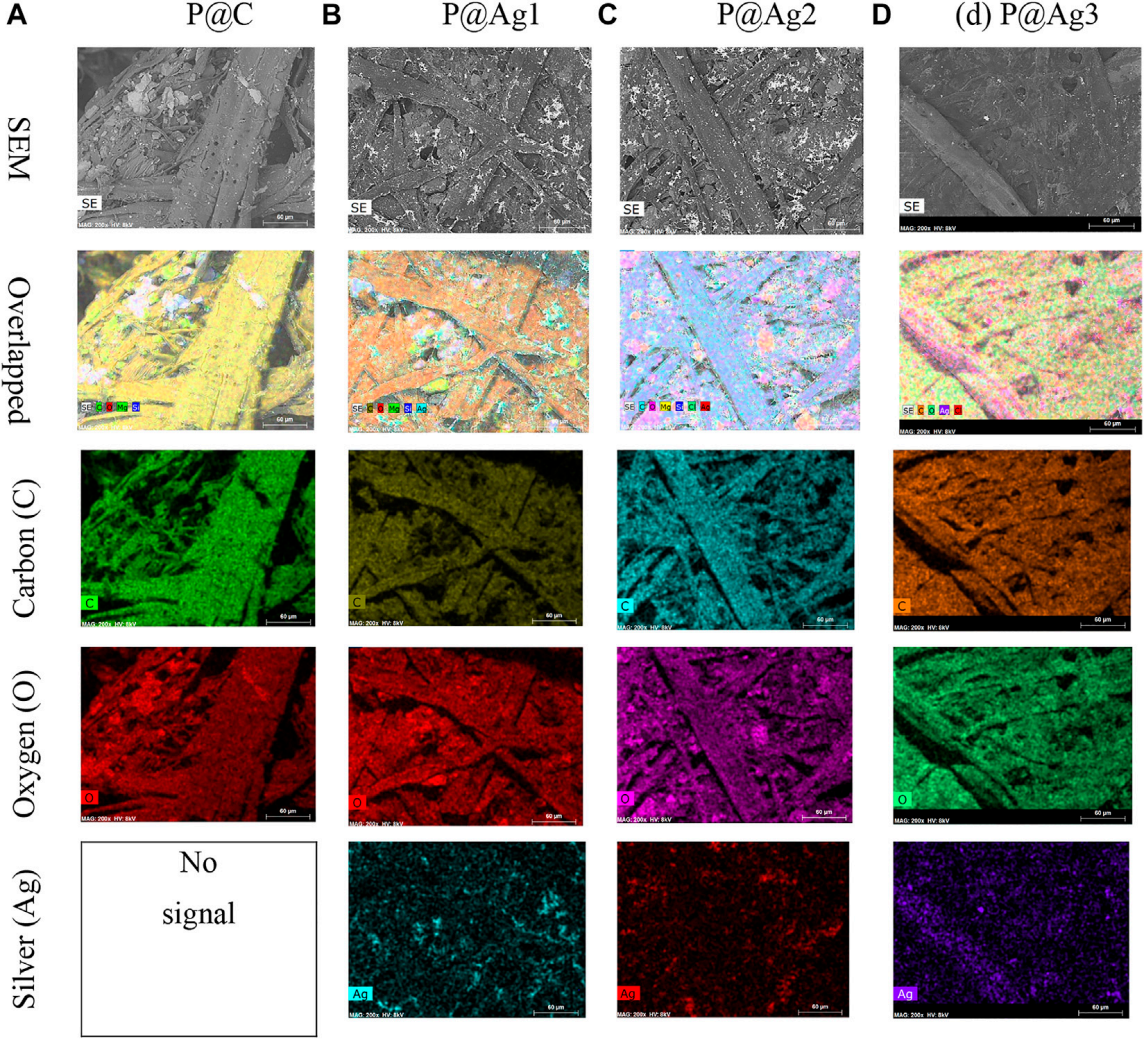
Elemental mapping images of control and nanosilver deposited kraft paper samples: **(A)** P@C, **(B)** P@Ag1, **(C)** P@Ag2, and **(D)** P@Ag3.

Similar effects were also found in EDX and elemental mapping investigations. Because cellulosic paper is enriched with carbon, oxygen, and hydrogen, the presence of carbon and oxygen is easily observed with the dominance in EDX spectra for all the peaks. EDX analysis cannot detect inert gases like H; hence H was not observed by EDX analysis. However, the nanosilver-loaded samples showed an extra peak at 2.96 keV, Figure 4B2,C2,D2. The weight% of the nanoparticle-treated samples were 5.6, 9.92, and 10.32, respectively for 0.5, 1.5, and 2.5 mM silver precursor treated samples. The similar results of EDX analysis and mapping for nanosilver-treated samples also agree with other studies (V. Singh et al., 2015; P. Singh et al., 2016; Soshnikova et al., 2018). Additionally, EDX analysis is also in line with the XRF analysis. However, some impurities in negligible weight% were also noticed (Figure 4). Additionally, elemental mapping analysis further confirmed the detection of AgNPs (Figure 5). The uniform and homogeneous distributions of AgNPs are also apparent in the overlapped and SEM images of elemental mapping. Overall, the SEM, EDX, and elemental mapping studies further demonstrate the successful synthesis and attachments of metallic silver on the paper surfaces.

### 3.4 XRD analysis of the kraft paper materials

**FIGURE 6:**
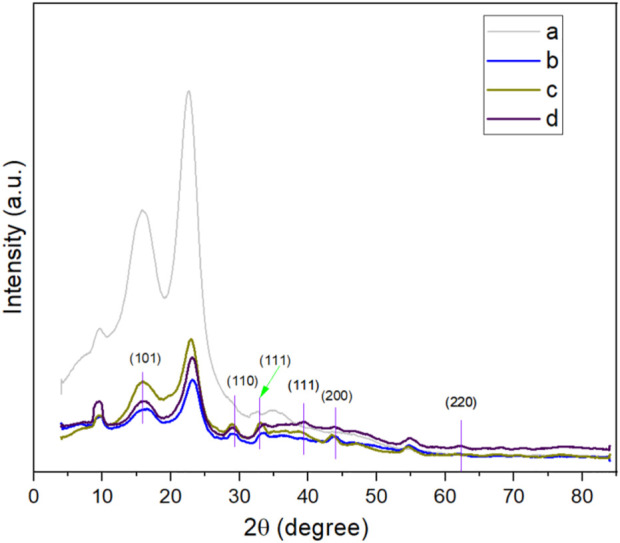
**(A)** XRD analysis of control and AgNP-treated kraft papers.

The effects of AgNP treated kraft papers were further examined by XRD analysis in terms of crystallinity of the materials (both control kraft paper and 0.5–2.5 mm AgNO_3_ treated kraft papers). Although cellulosic materials show a semi-crystalline nature, AgNPs show a crystalline nature (Ibrahim, 2015). The peaks located near 2θ = 16.7, 22.8, 34.6 (control kraft paper) are associated with the characteristics of 101, 200, and 040 reflections due to cellulose Ⅰ (Liu et al., 2012; Hajji et al., 2016). However, the peak intensities decreased after the AgNPs were incorporated into the papers. The peaks at 2θ = 38.6, 44.3, and 64.5° are related to (111), (200), (220), reflecting the presence of AgNPs in the paper materials (Figure 6). The control paper did not display any similar peaks. Some additional slight peaks were also observed at 28.83, 32.9, and 54.92° (according to Bragg peaks) indicating the presence of plant-based extracts used for AgNPs synthesis. The extracts functioned as the reducing and stabilizing agent (Roopan et al., 2013). Therefore, XRD analysis also further confirms the successful deposition and binding of AgNPs with the paper materials.

### 3.5 Colorimetric studies of the products

The color of manufactured products is a key factors in consumer inclinations and product demand. The CIE Lab system is also widely used for assessing both traditional and nanosilver-treated color properties in terms of L*, a*, and b* values. Table 2 shows the color coordinates of the AgNP-treated products. The AgNP deposited kraft papers display versatile, magnificent colors ranging from light yellow to dark brown, whereas the control kraft paper exhibits nearly white tones. The color darkness/lightness and color strength (K/S) values demonstrate an increasing trend with the increase in nanosilver precursor in the nanocolloid system. The LSPR (localized surface plasmon resonance) optical properties of the metallic silver is the cause of the brilliant coloration effects on the sample surfaces (Aziz et al., 2019; Amirjani et al., 2020). The control paper sample had the highest L* values with 87.45, whereas 0.5 mm AgNO_3_ treated samples had a value of 37.3, 1.0 mm AgNO_3_ treated samples had a value of 34.05, and 2.5 mm AgNO_3_ treated samples had a value of 31.77. The higher r L* value, the lighter the sample in terms of color. Lower L* values indicate darker samples. It seems the increased loading of nanoparticles makes the sample darker. Although the samples initially showed slightly reddish/yellow colors, they turned reddish/brownish with the increase in nanosilver loading. A similar trend is also noticed in K/S values. The 0.5 mm AgNO_3_ treated sample displayed a strength of 3.89, which is 35% less than the 2.5 mm silver precursor treated samples (5.98) and 18% less than 1.5 mm silver precursor sample, which had a strength of only 4.93. Additionally, all the a* and b* values present positive integers in varying ranges, demonstrating the appearance of multiple colors. The colorimetric data also agree with the visual assessment of the samples (Figure 3). The characteristics discussed concur with other research studies (González et al., 2014; Y. Zhou et al., 2018).

**TABLE 2:** Colorimetric data for control and AgNP-treated kraft papers.

Specimens	(A) Nanotreatment recipe	(B) Colorimetric data				(C) Fastness values (color)
	AgNO_3_ (mM)/fruit extract (MW in % (v/v))	L*	a*	b*	K/S	LF
P@C	0/0	87.45	−1.11	11.44	0.05	‒
P@Ag1	0.5/4.0	37.3	16.35	39.11	3.89	6–7
P@Ag2	1.5/4.0	34.05	15.46	39.42	4.93	5–6
P@Ag3	2.5/4.0	31.77	13.59	31.06	5.98	5
R^2^	‒	0.99	0.98	0.99	0.98	‒

The current study also examined the fastness properties of the samples to understand product color stability after AgNPs deposition. We found that although less nanosilver coating provides lower color strengths, color stability becomes higher compared to higher nanoparticle-loaded samples (Table 2). Therefore, 0.5 mm AgNPs loaded samples displayed light fastness properties of 6 (demonstrating “Very Good” to “Excellent”), and 2.5 mm AgNPs loaded samples provided light fastness properties of 5, which is only “Good”. However, the 1.5 mm AgNPs loaded sample presented light fastness properties within 5-6, which are “Good” and “Very Good” ratings. It seems the fastness properties of the nanoparticle-treated colored samples can also be tuned by controlling the silver precursor concentrations in the nanocolloids.

### 3.6 Statistical analysis of the colorimetric data

Color properties were investigated further via R^2^ values. These investigations found that the increased nanosilver loading also plays a significant role in the enhanced colorimetric properties. As seen in Table 2, the R^2^ values are equal to or greater than 0.99, demonstrating a strong correlation between the loaded nanoparticles in the kraft paper samples and all the colorimetric characteristics (K/S, L*, a*, and b*). The one-way ANOVA test with the adopted Newman-Keuls test further confirms these results (Tables 3–7). Except for two colorimetric data (a* and b*), all the values display p-values less than 0.05, especially for the XRF test, K/S, and L* values. Three cases revealed *p*-values higher than 0.05 both for a* and b* characteristics, which are in bold fonts. However, statistical data analysis summarizes that increased concentrations of AgNO_3_ can increase the nanosilver development over the kraft paper materials. Additionally, the developed nanoparticles have a significant influence on their colorimetric properties. Other researchers have also reported some statistical models for nanosilver-treated products (Ahmadi et al., 2018; Veeraraghavan et al., 2021). However, to the best of our knowledge, no studies have reported on the colorimetric modelling in terms of the Newman-Keuls test covered in the present study. Therefore, comparative analysis could be performed especially for statistical analysis.

**TABLE 3:** Newman-Keul test for XRF test values in terms of AgNO3 loading for P@Ag1, P@Ag2, and P@Ag3 samples.

Specimen	1	2	3	4
P@C		0.00022	0.00020	0.00023
P@Ag1	0.00022		0.00022	0.00020
P@Ag2	0.00020	0.00022		0.00024
P@Ag3	0.00023	0.00020	0.00024	

**TABLE 4:** Newman-Keul test for K/S values in terms of nanosilver loading for P@Ag1, P@Ag2, and P@Ag3 samples.

Specimen	1	2	3	4
P@C		0.00022	0.000201	0.000231
P@Ag1	0.00022		0.015598	0.00020
P@Ag2	0.00020	0.015598		0.00024
P@Ag3	0.00023	0.00020	0.00024	

**TABLE 5:** Newman-Keul test for L* values in terms of nanosilver loading for P@Ag1, P@Ag2, and P@Ag3 samples.

Specimen	1	2	3	4
P@C		0.00022	0.00020	0.00023
P@Ag1	0.00022		0.01316	0.00153
P@Ag2	0.00020	0.01317		0.04561
P@Ag3	0.00023	0.00153	0.04561	

**TABLE 6:** Newman-Keul test for a* values in terms of nanosilver loading for P@Ag1, P@Ag2, and P@Ag3 samples.

Specimen	1	2	3	4
P@C		0.00023	0.00020	0.00022
P@Ag1	0.000231		0.11573	0.05031
P@Ag2	0.000201	0.11573		0.30766
P@Ag3	0.00022	0.05031	0.30766	

**TABLE 7:** Newman-Keul test for b* values in terms of nanosilver loading for P@Ag1, P@Ag2, and P@Ag3 samples.

Specimen	1	2	3	4
P@C		0.00030	0.00037	0.00064
P@Ag1	0.00030		0.23506	0.04371
P@Ag2	0.00037	0.23506		0.134,370
P@Ag3	0.00064	0.04371	0.13437	

### 3.7 Mechanical properties of the control and coated kraft papers

Together with coloration characteristics, mechanical properties are a significant factor in packaging products. Mechanical properties like tensile index and bursting strength were investigated and are shown in (Table 8) in lengthwise/machine directions. The control papers displayed tensile and bursting indexes of 55.21 ± 1.23 and 2.62 ± 0.08 Nm/g, respectively. However, both properties had increasing trends with the increase in nanosilver loading. P@Ag1 presented tensile and bursting indexes of 57.11 ± 1.23 and 2.78 ± 0.14 Nm/g, P@Ag2 had 58.8 ± 2.05 and 2.97 ± 0.212 Nm/g, and P@Ag3 exhibited 61 ± 1.93 and 3.12 ± 0.05 Nm/g, respectively. It seems AgNPs played a great role not only in enhancing the brilliant appearance of the papers, but in increasing mechanical properties. Other studies found similar results for the tensile and bursting properties of kraft paper substrates (Nokkrut et al., 2019). The results (both tensile and bursting index) further demonstrate the potential for packaging products with improved strength.

**TABLE 8:** Tensile and bursting properties of the control and coated kraft papers. Results are shown here in Mean± standard deviations.

Mechanical properties	P@C	P@Ag1	P@Ag2	P@Ag3
Tensile Index	55.21 ± 1.23	57.11 ± 1.23	58.8 ± 2.05	61 ± 1.93
Bursting index	2.62 ± 0.08	2.78 ± 0.14	2.97 ± 0.212	3.12 ± 0.05

### 3.8 FTIR properties of the control and coated kraft papers

**FIGURE 7:**
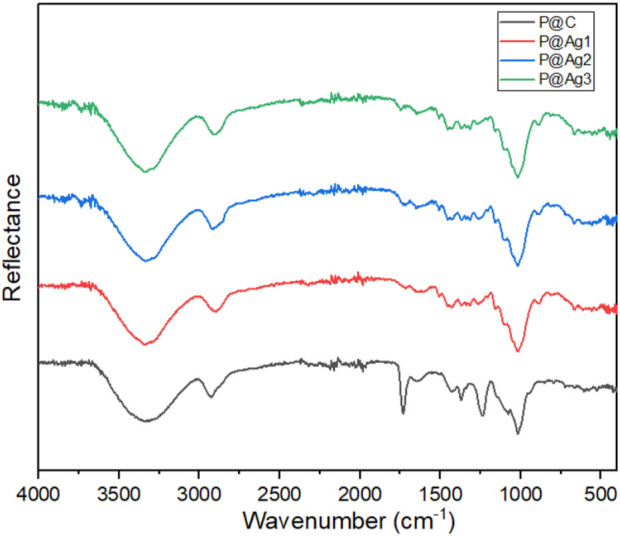
FTIR analysis of control and AgNP coated kraft papers.

The chemical interactions of the control and AgNPs-treated kraft paper products were further investigated using FTIR characterizations to understand the effects of nanoparticles on cellulosic materials structure. The peaks clearly display the typical characteristics of cellulosic materials as shown in Figure 7. This finding agrees with other research studies (Fan et al., 2012; Rac-Rumijowska et al., 2019). The broad peaks around 3,330 cm^−1^ are attributed to the presence of the -OH group while 2,920, 1,425, and 1,365 cm^−1^ are related to the C-H group. The peaks around 1,636 cm^−1^ provide information concerning the absorbed water. The absorption bands at 1,230 and 1,075 cm^−1^ confirm the presence of C-OH groups. Furthermore, the peaks at 1,635 cm^−1^ indicate the presence of stretching vibrations of the CH_2_ bond. The small peak at 895 cm^−1^ is related to the C-O-C bond. However, the similar peaks are still visible after green AgNPs coating. The deposited AgNPs might be distributed in the cellulosic molecules of paper in only metallic form and potentially as a metalorganic element (Rac-Rumijowska et al., 2019). However, the peaks around 1,398 and 1,125 cm^−1^ in treated samples could be associated with the metal-oxygen and metal-OH bond, which are not seen in control kraft papers (Azari et al., 2015; Rac-Rumijowska et al., 2019).

### 3.9 UV-protection properties of control and coated kraft papers

Food packaging paper materials are also frequently exposed to sunlight; hence, the UPF, UVA, and UVB properties were assessed further. The UPF characteristics demonstrate the time that kraft paper materials can be exposed to light without any potential damage. Values under 15 are considered lower, 15 to 24 as good, 24 to 39 as very good, and over 40 is considered an excellent shield against UV-rays, confirming that a significant amount of UV light is absorbed. However, UV protection depends on many factors. Without any special treatment, attaining UV shielding characteristics is unfeasible. Therefore, the kraft papers were treated with AgNPs to increase UV protection capabilities. The control paper showed a UPF value of only 20.99, a UVA value of 8.49, and a UVB value of 3.64. On the other hand, the AgNP-treated products revealed a significant improvement. All of the UPF values were more than 40, implying significant UV protection capabilities of the papers (42.39 for 0.5 mm, 48.48 for 1.5 mm, and 54.5 for 2.5 mm silver precursor treated samples). The increase in nanosilver concentrations also displayed an increasing trend in UPF values (Table 9). The results discussed here also agree with different cellulosic substrates treated with AgNPs (Haslinger et al., 2019). Therefore, the overall results confirm the strong UV blocking capabilities of the AgNPs treated papers.

**TABLE 9:** UV-protection properties of control and AgNP coated papers.

Test specimens	UPF	UVA (%)	UVB (%)
P@C	20.99	8.49	3.64
P@Ag1	42.39	2.73	1.99
P@Ag2	48.48	2.06	1.77
P@Ag3	54.5	1.36	1.56

### 3.10 Thermal properties of control and coated kraft papers

**FIGURE 8:**
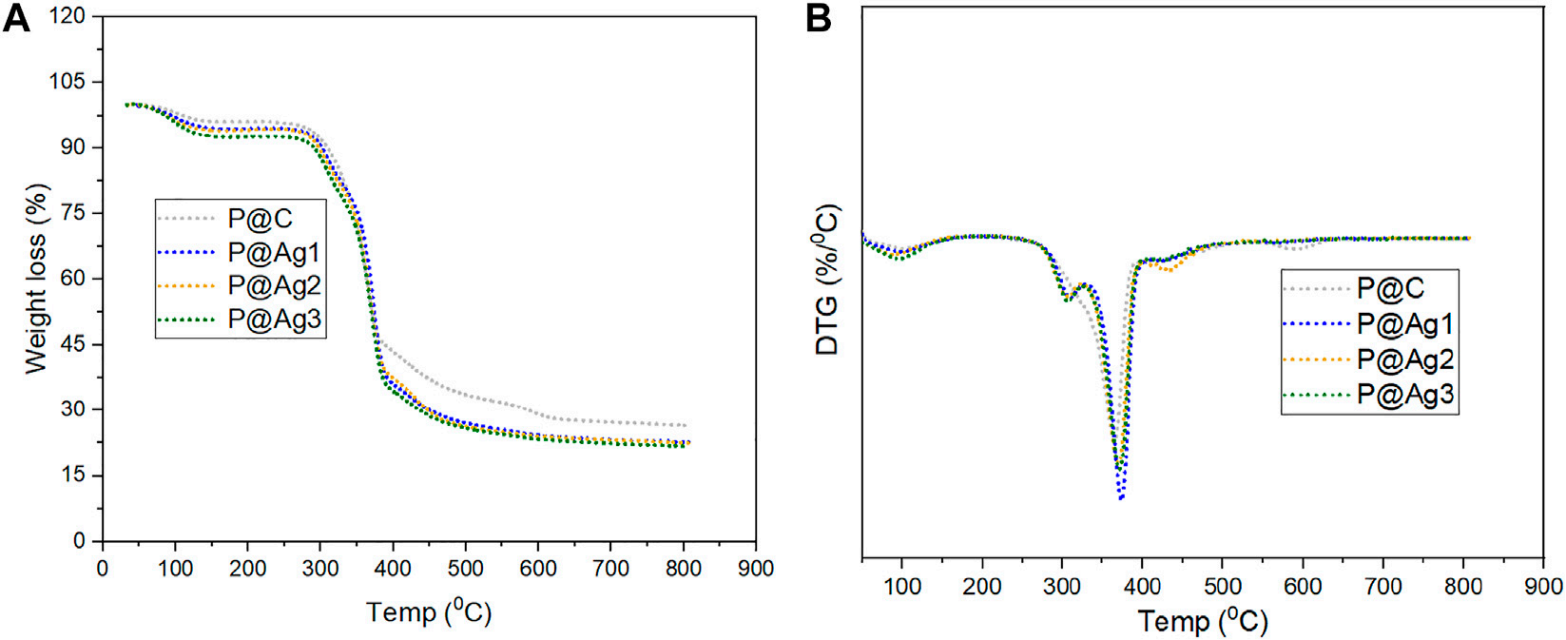
Thermal properties analysis of control and AgNP coated kraft papers: **(A)** TGA analysis and **(B)** DTG analysis.

The thermal performances of the control and AgNPs-treated products were also investigated via the thermogram records (Figure 8A,B). An initial weight loss is observed until 100°C (Lu et al., 2015), which demonstrates the evaporation of adsorbed water in the control and coated kraft papers. A major weight loss occurs within the range of 280–390°C. This loss can be attributed to the degradation of major molecular chains from cellulosic kraft papers (Q. Zhou et al., 2017). The weight loss up to 400°C corresponds to the breakdown of cellulose. However, a slight deviation in thermal stability is noticed for AgNPs-treated test specimens compared to the control kraft paper materials, although nanosilver loading increased the mechanical properties and UV-shielding capabilities. This phenomenon also conforms with other studies for *Tinospora cordifolia* extract-mediated cotton fibers functionalization with AgNPs (Gollapudi et al., 2020). In DTG analysis, peaks around 300°C demonstrate hemicellulose degradation, and the peaks around 370°C correspond to cellulose from kraft fiber materials. Overall, a successful binding of AgNPs with the kraft paper facilitated the changes in thermal properties.

### 3.11 Assessing safety aspects of the AgNP-treated products

Previously, chemical-based synthesis protocols were used to synthesize metallic silvers because the green synthesis protocol was not explored much. Toxicity was a big challenge for chemically modified nanosilver-treated products (Levard et al., 2012). However, the green synthesis protocol provides a new route toward safe, toxin-free metallic silver (Jadoun et al., 2021). The treated kraft papers were washed with distilled water five times, and then the concentrations of remaining AgNPs were measured again using an XRF instrument. The values found were 262 (2), 780 (3), and 992 5) PPM, respectively. These were calculated to understand the release% of Ag^+^ from the AgNPs-treated kraft papers. The calculations found that the 0.5 mm AgNO_3_ treated samples released 2% Ag^+^, whereas 1.5 and 2.5 mm AgNO_3_ treated samples released 3 and 5% Ag^+^, respectively. A recent study reported that AgNP-treated products could be considered safe materials without any cytotoxic effects on human health in cases in which the release of silver is less than 10 PPM (Suárez et al., 2010); the products the current study reports are also in line with these requirements.

## 4 Conclusion

The present study demonstrated an eco-friendly, one-step, efficient, facile, and simple method for AgNPs deposition on kraft paper using *in situ* synthesis protocol. The greenly synthesized AgNPs coatings were successfully performed by varying the concentrations of silver precursor in the presence of *Cephalotaxus harringtonia* as a stabilizing agent. Interestingly, we noticed that the coated NPs are very stable, and the release of metallic silver from coated kraft paper surfaces is minimal. It seems the coating has lifelong durability on paper surfaces. The XRF analysis revealed a significant presence of metallic silver with 268 ± 7, 805 ± 14, and 1,045 ± 16 PPM. Morphological images of deposited surfaces also exhibited the explicit presence of AgNPs. Moreover, the EDX analysis and elemental mapping of the AgNP-coated products displayed and confirmed the metallic silver presence on the sample surfaces. Moreover, the colorimetric data also showed the development of colorful paper products that can be used for food packaging materials. Mechanical tests demonstrated significant performance regarding the tensile and bursting index. The UPF factor of nanosilver coated papers had values higher than 40, indicating successful shielding against sunlight. The FTIR analysis, XRD test, TGA/DTG analysis, mechanical properties, and statistical analysis further confirmed the successful depositions of greenly synthesized AgNPs on the paper surfaces, which can be used for packaging applications.

## Data Availability

The original contributions presented in the study are included in the article/supplementary material, further inquiries can be directed to the corresponding author.

## References

[B1] Abou El-Nour K. M. Eftaiha A. Al-Warthan A. Ammar R. A. (2010). Synthesis and applications of silver nanoparticles. Arab. J. Chem. 3, 135–140. 10.1016/j.arabjc.2010.04.008

[B2] Ahmadi O. Jafarizadeh-Malmiri H. Jodeiri N. (2018). Eco-friendly microwave-enhanced green synthesis of silver nanoparticles using Aloe vera leaf extract and their physico-chemical and antibacterial studies. Green process. Synth. 7, 231–240. 10.1515/gps-2017-0039

[B3] Aladpoosh R. Montazer M. Samadi N. (2014). *In situ* green synthesis of silver nanoparticles on cotton fabric using Seidlitzia rosmarinus ashes. Cellulose 21, 3755–3766. 10.1007/s10570-014-0369-1

[B4] Amirjani A. Firouzi F. Haghshenas D. F. (2020). Predicting the size of silver nanoparticles from their optical properties. Plasmonics 15, 1077–1082. 10.1007/s11468-020-01121-x

[B28] Aryan Ruby Mehata M. S. (2021). Green synthesis of silver nanoparticles using Kalanchoe pinnata leaves (life plant) and their antibacterial and photocatalytic activities. Chem. Phys. Lett. 778, 138760. 10.1016/j.cplett.2021.138760

[B5] Ashok B. Obi Reddy K. Yorseng K. Rajini N. Hariram N. Siengchin S. (2018). Modification of natural fibers from Thespesia lampas plant by *in situ* generation of silver nanoparticles in single-step hydrothermal method. Int. J. Polym. Analysis Charact. 23, 509–516. 10.1080/1023666X.2018.1486270

[B6] Azari A. Kalantary R. R. Ghanizadeh G. Kakavandi B. Farzadkia M. Ahmadi E. (2015). Iron–silver oxide nanoadsorbent synthesized by co-precipitation process for fluoride removal from aqueous solution and its adsorption mechanism. RSC Adv. 5, 87377–87391. 10.1039/C5RA17595J

[B7] Aziz S. B. Hussein G. Brza M. A. J. Mohammed S. T. Abdulwahid R. Raza Saeed S. (2019). Fabrication of interconnected plasmonic spherical silver nanoparticles with enhanced localized surface plasmon resonance (LSPR) peaks using quince leaf extract solution. Nanomaterials 9, 1557. 10.3390/nano9111557 PMC6915396 31684041

[B8] Cittrarasu V. Kaliannan D. Dharman K. Maluventhen V. Easwaran M. Liu W. C. (2021). Green synthesis of selenium nanoparticles mediated from Ceropegia bulbosa Roxb extract and its cytotoxicity, antimicrobial, mosquitocidal and photocatalytic activities. Sci. Rep. 11, 1032–1115. 10.1038/s41598-020-80327-9 33441811 PMC7806947

[B9] Da-Cheng H. Hou X. D. Gu X. J. Xiao P. G. Ge G. B. (2021). Ethnopharmacology, chemodiversity, and bioactivity of Cephalotaxus medicinal plants. Chin. J. Nat. Med. 19, 321–338. 10.1016/S1875-5364(21)60032-8 33941338

[B10] Eustis S. El-Sayed M. A. (2006). Why gold nanoparticles are more precious than pretty gold: Noble metal surface plasmon resonance and its enhancement of the radiative and nonradiative properties of nanocrystals of different shapes. Chem. Soc. Rev. 35, 209–217. 10.1039/B514191E 16505915

[B11] Fan M. Dai D. Huang B. (2012). “Fourier transform infrared spectroscopy for natural fibres,” in Fourier transform-materials analysis (London, UK: IntechOpen), 45–68.

[B12] Gollapudi V. R. Mallavarapu U. Seetha J. Akepogu P. Amara V. R. Natarajan H. (2020). *In situ* generation of silver and silver oxide nanoparticles on cotton fabrics using Tinospora cordifolia as bio reductant. SN Appl. Sci. 2, 508–510. 10.1007/s42452-020-2331-1

[B13] González A. Noguez C. Beranek J. Barnard A. S. (2014). Size, shape, stability, and color of plasmonic silver nanoparticles. J. Phys. Chem. C 118, 9128–9136. 10.1021/jp5018168

[B14] Hajji L. Boukir A. Assouik J. Pessanha S. Figueirinhas J. L. Carvalho M. L. (2016). Artificial aging paper to assess long-term effects of conservative treatment. Monitoring by infrared spectroscopy (ATR-FTIR), X-ray diffraction (XRD), and energy dispersive X-ray fluorescence (EDXRF). Microchem. J. 124, 646–656. 10.1016/j.microc.2015.10.015

[B15] Haslinger S. Ye Y. Rissanen M. Hummel M. Sixta H. (2019). Cellulose fibers for high-performance textiles functionalized with incorporated gold and silver nanoparticles. ACS Sustain. Chem. Eng. 8, 649–658. 10.1021/acssuschemeng.9b06385

[B16] Heera P. Shanmugam S. Ramachandran S. (2015). Green synthesis of copper nanoparticle using *Gymnema sylvestre* by different solvent extract. Int. J. Curr. Res. Acad. Rev. 3, 268–275.

[B17] Ibrahim H. M. (2015). Green synthesis and characterization of silver nanoparticles using banana peel extract and their antimicrobial activity against representative microorganisms. J. Radiat. Res. Appl. Sci. 8, 265–275. 10.1016/j.jrras.2015.01.007

[B18] Islam M. A. Jacob M. V. Antunes E. (2021). A critical review on silver nanoparticles: From synthesis and applications to its mitigation through low-cost adsorption by biochar. J. Environ. Manag. 281, 111918. 10.1016/j.jenvman.2020.111918 33433370

[B19] Jadoun S. Arif R. Jangid N. K. Meena R. K. (2021). Green synthesis of nanoparticles using plant extracts: A review. Environ. Chem. Lett. 19, 355–374. 10.1007/s10311-020-01074-x

[B20] Kedi P. B. E. Eya'ane Meva F. Kotsedi L. Nguemfo E. L. Bogning Zangueu C. Ntoumba A. A. (2018). Eco-friendly synthesis, characterization, *in vitro* and *in vivo* anti-inflammatory activity of silver nanoparticle-mediated Selaginella myosurus aqueous extract. Int. J. Nanomedicine 13, 8537–8548. 10.2147/ijn.s174530 30587976 PMC6296690

[B21] Khatami M. Iravani S. Varma R. S. Mosazade F. Darroudi M. Borhani F. (2019). Cockroach wings-promoted safe and greener synthesis of silver nanoparticles and their insecticidal activity. Bioprocess Biosyst. Eng. 42, 2007–2014. 10.1007/s00449-019-02193-8 31451901

[B22] Kulkarni V. Kulkarni P. (2013). Green synthesis of copper nanoparticles using *Ocimum sanctum* leaf extract. Int. J. Chem. Stud. 1, 1–4.

[B23] Levard C. Hotze E. M. Lowry G. V. Brown G. E. (2012). Environmental transformations of silver nanoparticles: Impact on stability and toxicity. Environ. Sci. Technol. 46, 6900–6914. 10.1021/es2037405 22339502

[B24] Li Y.-Z. Wang Y. T. Zhao C. X. Jing Q. X. Jiang C. Y. Lin B. (2021). Cephalotaxine-type alkaloids with antiproliferation effects from the branches and leaves of *Cephalotaxus fortunei* var. alpina. Fitoterapia 155, 105037. 10.1016/j.fitote.2021.105037 34536534

[B25] Liu Y. Thibodeaux D. Gamble G. Bauer P. VanDerveer D. (2012). Comparative investigation of Fourier transform infrared (FT-IR) spectroscopy and X-ray diffraction (XRD) in the determination of cotton fiber crystallinity. Appl. Spectrosc. 66, 983–986. 10.1366/12-06611 22800914

[B26] Lu Z. Xiao J. Wang Y. Meng M. (2015). *In situ* synthesis of silver nanoparticles uniformly distributed on polydopamine-coated silk fibers for antibacterial application. J. Colloid Interface Sci. 452, 8–14. 10.1016/j.jcis.2015.04.015 25909867

[B27] Maghimaa M. Alharbi S. A. (2020). Green synthesis of silver nanoparticles from Curcuma longa L. and coating on the cotton fabrics for antimicrobial applications and wound healing activity. J. Photochem. Photobiol. B Biol. 204, 111806. 10.1016/j.jphotobiol.2020.111806 32044619

[B29] Mohammadalinejhad S. Almasi H. Esmaiili M. (2019). Simultaneous green synthesis and *in-situ* impregnation of silver nanoparticles into organic nanofibers by Lythrum salicaria extract: Morphological, thermal, antimicrobial and release properties. Mater. Sci. Eng. C 105, 110115. 10.1016/j.msec.2019.110115 31546384

[B30] Nechita P. Roman Iana-Roman M. (2020). Review on polysaccharides used in coatings for food packaging papers. Coatings 10, 566. 10.3390/coatings10060566

[B31] Nokkrut B.-o. Pisuttipiched S. Khantayanuwong S. Puangsin B. (2019). Silver nanoparticle-based paper packaging to combat black anther disease in orchid flowers. Coatings 9, 40. 10.3390/coatings9010040

[B32] Parvataneni R. (2020). Biogenic synthesis and characterization of silver nanoparticles using aqueous leaf extract of Scoparia dulcis L. and assessment of their antimicrobial property. Drug Chem. Toxicol. 43, 307–321. 10.1080/01480545.2018.1505903 30915859

[B33] Rac-Rumijowska O. Maliszewska I. Fiedot-Tobola M. Karbownik I. Teterycz H. (2019). Multifunctional nanocomposite cellulose fibers doped *in situ* with silver nanoparticles. Polymers 11, 562. 10.3390/polym11030562 PMC6473758 30960546

[B34] Ramadan M. A. Sharawy S. Elbisi M. Ghosal K. (2020). Eco-friendly packaging composite fabrics based on *in situ* synthesized silver nanoparticles (AgNPs) & treatment with chitosan and/or date seed extract. Nano-Structures Nano-Objects 22, 100425. 10.1016/j.nanoso.2020.100425

[B35] Roopan S. M. Rohit Madhumitha G. Rahuman A. Kamaraj C. Bharathi A. (2013). Low-cost and eco-friendly phyto-synthesis of silver nanoparticles using Cocos nucifera coir extract and its larvicidal activity. Ind. Crops Prod. 43, 631–635. 10.1016/j.indcrop.2012.08.013

[B36] Singh P. Kim Y. J. Yang D. C. (2016). A strategic approach for rapid synthesis of gold and silver nanoparticles by *Panax ginseng* leaves. Artif. Cells Nanomed. Biotechnol. 44, 1949–1957. 10.3109/21691401.2015.1115410 26698271

[B37] Singh V. Ankita S. Nitin W. (2015). Biosynthesis of silver nanoparticles by plants crude extracts and their characterization using UV, XRD, TEM and EDX. Afr. J. Biotechnol. 14, 2554–2567. 10.5897/AJB2015.14692

[B38] Soshnikova V. Kim Y. J. Singh P. Huo Y. Markus J. Ahn S. (2018). Cardamom fruits as a green resource for facile synthesis of gold and silver nanoparticles and their biological applications. Artif. Cells Nanomed. Biotechnol. 46, 108–117. 10.1080/21691401.2017.1296849 28290213

[B39] Suárez M. Esteban-Tejeda L. Malpartida F. Fernandez A. Torrecillas R. Moya J. (2010). Biocide activity of diatom-silver nanocomposite. Mat. Lett. 64, 2122–2125. 10.1016/j.matlet.2010.06.061

[B40] Veeraraghavan V. P. Periadurai N. D. Karunakaran T. Hussain S. Surapaneni K. M. Jiao X. (2021). Green synthesis of silver nanoparticles from aqueous extract of Scutellaria barbata and coating on the cotton fabric for antimicrobial applications and wound healing activity in fibroblast cells (L929). Saudi J. Biol. Sci. 28, 3633–3640. 10.1016/j.sjbs.2021.05.007 34220213 PMC8241602

[B41] Zhou Q. Lv J. Ren Y. Chen J. Gao D. Lu Z. (2017). A green *in situ* synthesis of silver nanoparticles on cotton fabrics using Aloe vera leaf extraction for durable ultraviolet protection and antibacterial activity. Text. Res. J. 87, 2407–2419. 10.1177/0040517516671124

[B42] Zhou Y. Tang R. C. (2018). Facile and eco-friendly fabrication of colored and bioactive silk materials using silver nanoparticles synthesized by two flavonoids. Polymers 10, 404. 10.3390/polym10040404 PMC6415457 30966439

[B43] Zuas O. Hamim N. Sampora Y. (2014). Bio-synthesis of silver nanoparticles using water extract of Myrmecodia pendan (Sarang Semut plant). Mat. Lett. 123, 156–159. 10.1016/j.matlet.2014.03.026

